# The Influence of Different Types of Outdoor Access on Dairy Cattle Behavior

**DOI:** 10.3389/fvets.2020.00257

**Published:** 2020-05-13

**Authors:** Anne-Marieke C. Smid, Daniel M. Weary, Marina A. G. von Keyserlingk

**Affiliations:** Animal Welfare Program, Faculty of Land and Food Systems, University of British Columbia, Vancouver, BC, Canada

**Keywords:** animal welfare, pasture, bedded pack, exercise lot, free range

## Abstract

Pasture access for dairy cows is highly valued both by cows and the public at large. When pasture access is not feasible, farmers can provide cows with alternative forms of outdoor access, such as an outdoor bedded pack, that may be easier to implement on some farms. We reviewed the literature on how lying, standing, walking, feeding, social, and estrus behaviors are influenced by pasture and other types of outdoor areas. Pasture allows the expression of grazing and can facilitate the expression of lying, standing, walking, and estrus behaviors. In addition, pasture can decrease the number of negative social interactions between cows, likely because more space per cow is provided than what is normally available indoors. The provision of soft flooring and an open space in outdoor bedded packs appears to provide some benefits for lying, standing, and walking behavior and may also have positive effects on social behavior, especially with larger space allowances. The effects of an outdoor bedded pack on estrus behavior are less well-documented, but the provision of a standing surface that provides better footing than typically available indoors may promote estrus behavior. Alternative outdoor areas assessed to date appear to be less attractive for cows than pasture, perhaps because these areas do not provide the opportunity to graze. We encourage future research to investigate the importance of grazing for dairy cows. The motivation of dairy cows to access alternative outdoor areas should also be investigated. As cow preference for the outdoors depends on many factors, providing cows a choice may be of particular importance.

## Introduction

Pasture access for dairy cows is declining in many parts of the world, even though citizens from different countries view pasture as important [e.g., The Netherlands: (1); Germany: (2); Canada and the US: (3); Brazil: (4)]. Collectively these studies indicate that people value access to natural elements for cows such as fresh air and sunshine and the ability to roam, i.e., elements that extend beyond the provision of pasture *per se*. There is also evidence that cows are highly motivated to access pasture (5). As such, several Nordic European countries have implemented regulations that require farms to provide dairy cows with access to pasture for specified periods of time. Organic standards in many parts of the world also regulate access to pasture, at least for part of the year (6). However, in many parts of the world pasture access is not regulated. When farm size increases, pasture access may also be difficult to implement (7). An alternative to pasture is providing cows access to an outdoor loafing area (i.e., an open area with concrete or other hard flooring) or to an outdoor bedded open pack (i.e., an open area with a soft flooring). Given that these alternative outdoor options generally require less space per cow than pasture and are less subject to damage from cow traffic than pasture, they may be easier to implement on some farms. However, little is known about how alternative outdoor areas influence cow behavior. The aim of this review was to critically assess the scientific literature to understand how key behaviors (lying, standing, walking, feeding, social, and estrus) are influenced by pasture and other types of outdoor area. We also identify gaps in knowledge, especially regarding the use of alternative outdoor areas. Where applicable we draw upon research that investigates cow preference and motivation, as this evidence is especially helpful for drawing inferences regarding the importance of outdoor access to cows (8). We recognize that many aspects of dairy cattle welfare are influenced by pasture and other types of outdoor access, including health and production measures but this is beyond the scope of this review [see (9) for more information]. For the remainder of this review, we will focus on the influence of different types of outdoor access on dairy cattle behavior.

## Outdoor Access for Dairy Cattle

In this section, we provide an overview of pasture and alternative types of outdoor access used on dairy farms in those regions where we have been able to find reliable data (Europe, Australia, New Zealand, the United States and Canada). We distinguish between pasture (i.e., an outdoor area with grassland that allows for grazing) and alternative outdoor environments (i.e., any type of outdoor area that has some sort of alternative flooring to grassland, such as concrete or bedding of some sort). Pasture and alternative outdoor areas provide cows with access to the outdoors, but the outdoor environments differ in terms of size and many other features (most notably pasture allows cows the opportunity to graze). This section builds upon the work presented in two recent reviews: one summarized the changes in the global dairy industry affecting dairy cattle health and welfare but did not examine pasture or outdoor access (10) and another focused upon pasture access for dairy cows, but not on alternative types of outdoor access (11).

### Europe

Information on pasture access for dairy cows in Europe is not collected in a systematic manner. As such, little is known about which cows are given access to pasture (e.g., young stock, lactating, dry cows, etc.) and duration of access (i.e., days per year and hours per day). In 2019, pasture access in Europe was estimated to range from 95–100% of dairy cows in Ireland, to <25% in Denmark, Poland, and Greece, with most other countries being intermediate [for an overview, please see (12)] (n.b. these figures do not distinguish between farms that provide cows free choice access to pasture from a barn vs. cows housed exclusively outdoors). Data from The Netherlands indicates that in 2018, 71% of the dairy cows aged 2 years and older had access to pasture (13); duration of pasture access was not specified. Regardless, the general trend in the majority of European countries is that the number of farms providing cows with pasture access is declining (12). The exception being some of the nordic countries, such as Sweden, Norway, and Finland, that have implemented regulations requiring farms to provide dairy cows access to pasture for specified periods (12). For example, in Sweden dairy cows must be given pasture access a minimum of 6 h/d, for 60–120 d/y, depending on the region (14). These regulations are based on the assumption that pasture provides cows with an environment in which they can better express natural behaviors such as grazing^1^. Similar to pasture access, there are limited data regarding the percentage of farms in Europe that use alternative outdoor areas. To our knowledge the only available scientific information comes from the 2015 European Food Safety Association (EFSA) report (15) detailing that 3 out of 124 small-scale/non-conventional farms in the convenience sample stated that they used an alternative outdoor area.

### Australia and New Zealand

Data collected in 2016 showed that about 99% of Australian dairy farms provided cows pasture access. The large majority (89%) kept cows on pasture year-round; 6% of the farms kept cows on pasture during most of the year but also provided supplementary feed (i.e., partial mixed ration) on an outdoor feed pad; 3% of the farms kept their cows on pasture for less than 9 m/y with a partial mixed ration provided on an outdoor feed pad, the latter two used some type of indoor housing or sheds the rest of the year [(16); personal communication]. Alternatives to pasture are commonly referred to as permanent feed pads with the majority of these using concrete flooring; temporary feed pads also exist and are generally differentiated from the permanent feed pads in that they have either a dirt or rubble (i.e., crushed rock and other materials with a range of particle sizes that can be compressed) base (17).

It is thought that more than 99% of dairy farms in New Zealand provide pasture access during some time or during the full year (DairyNZ, personal communication). Approximately one quarter of farms have an off-paddock system (i.e., an area that cows can be kept on during adverse weather conditions, or to reduce feed wastage) available on the farm. Of the farms using an off-paddock system, 81% do not provide any form of cover. Generally, the lying area comprises at least 80% of the total off-paddock surface area; concrete, gravel, and wood-chips are the most common form of surface material; the remaining area is often a concrete feed pad with feed through [(18); personal communication].

### United States and Canada

Pasture-based dairy farming was once the norm in the United States (19), but data from 2013 show that pasture is used as the primary system for fewer than 3% of lactating cows and for 5.0% of dry cows (20). A total of 19.9% and 34.0% of lactating and dry cows, respectively, had some pasture access (21). Approximately 26% of dairy cows in the US are housed in free-stalls with access to an open/dry lot and ~17% are housed in open/dry lots with or without access to a barn or shed (8.8 and 8.3%, respectively) (20). Although the majority of US dairy farms are still relatively small (i.e., in 2017, 74% of US dairy farms had <100 cows), 55% of all US dairy cows are housed on farms with >1,000 cows (22). As the percentage of lactating cows that have access to pasture decreases with increasing herd sizes, and an increasing volume of total milk production is produced by larger farms (23), the proportion of US dairy cows that have access to pasture is likely to decline.

**Figure 1:**
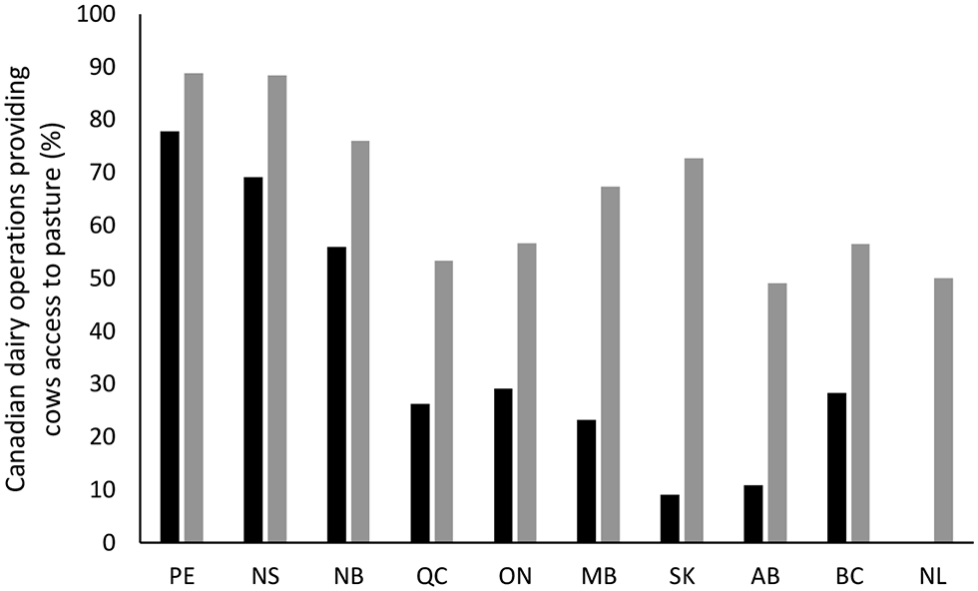
Percentage of operations that allow lactating (black bars) and dry (gray bars) cows access to pasture in Canada, by province (data should be viewed with caution as the survey only had a 9% response rate).

The National Dairy Study (2015) conducted in April–May 2015 [for detailed methodology, see (24)] contacted all dairy farms in Canada (*n* = 11,664) and obtained information regarding pasture usage. A total of 1,062 producers completed the full questionnaire (9% response rate). Of those farms that responded, 29.1% provided their lactating cows access to pasture with an approximate minimum average of (±SD) 20.5 ± 6.1 w/y. This corresponds to a total of 18.6% of lactating cows, although this number should be interpreted with caution, given that a significant proportion of all participants did not provide the number of lactating cows on their farm. Pasture use differed for lactating and dry cows and also by province (Figure 1). A total of 57.3% of farms provided dry cows pasture access, with an approximate minimum average of (±SD) 20.9 ± 9.5 w/y (farms often only provide dry cows access to pasture in spring and summer time, or weather permitting). This corresponds to a total of 49.2% of dry cows, although this number should again be interpreted with caution.

Collectively, the available evidence indicates that pasture use differs by region. Pasture usage is generally expected to decline in Europe and North America, driven by increases in farm size (7, 12). In addition to open lot dairies, some farms provide access to alternative types of outdoor environments such as bedded open packs or exercise lots (i.e., non-bedded areas with concrete or dirt as flooring), although the number of farms doing so appears to be limited outside of the US, Australia, and New Zealand. It is largely unknown what percentage of farms keep their cows outside as opposed to providing cows free choice access to the outdoors and some sort of indoor facility or covered area.

Space requirements for pasture, especially as herd sizes increase, may be one reason why pasture access is not provided. In addition, during some parts of the year pasture access may not be practical, for example during rainy seasons when the soil is subject to damage from cow traffic. This may be a reason why pasture access is more feasible in some parts of the world (e.g., New Zealand, Australia) than others (e.g., Canada and the USA). As will be discussed in the section on feeding behavior, some farmers believe that there are production benefits associated with zero-grazing systems. Farmer characteristics are important predictors of the degree of pasture access provided, as shown in studies with Irish (25) and German farmers (26). Given that the social factors influencing decisions regarding pasture access may differ by country, more research in this area is needed. In addition, most social science work has focussed on pasture access, and information regarding alternative outdoor areas is lacking.

## Dairy Cow Preference and Motivation for Outdoor Access

Preference testing requires animals to choose between two or more options (8). The “preferred” option is typically identified as the one that is chosen most often, consumed in the largest quantity, or where the majority of the available time is spent (27). Motivation testing investigates how hard an animal is willing to work to obtain access to a resource (28), i.e., a commodity or the opportunity for the animal to engage in a certain behavior (29). The stronger the motivation to access a resource, the more important that resource is thought to be for the animal (8, 30). Hence, welfare is thought to be more negatively affected if an animal is denied access to a resource for which it is highly motivated (8). Both preference and motivation tests may be affected by the animals' familiarity with the resource and can be influenced by the context in which the experiment is conducted (e.g., weather, time of day, etc.). These and other factors [for a comprehensive overview see (8)] should be taken into account when designing these types of experiments.

Several studies have shown that dairy cattle have a partial preference for pasture access (5, 31–35), with cows choosing to spend from 8% (34) to 72% (35) of their available time on pasture. Experience plays a role in dairy cattle preference for pasture. The cows used in the study of Charlton et al. (34) were reared indoors, potentially explaining why they only spent 8% of their time on pasture. Preference for pasture is influenced by environmental conditions, with high temperature-humidity index (THI) (31), and rainfall (31, 33, 34) decreasing the time spent outside. Cows prefer to spend time on pasture at night rather than during the day (31–33), possibly to avoid high solar radiation during the day (36). Several motivation tests have shown the importance of pasture access for cows, especially at night (5, 33). The quality of the indoor environment may also influence the value of outdoor access for dairy cattle. In a study by Falk et al. (37), cow preference for pasture was not influenced by the number of lying stalls available indoors (24, 16, 8, or 0 stalls per group of 24 cows), showing that even when overstocked cows preferred to be indoors for much of the day and on pasture at night. More research on how the indoor environment influences preference to be outdoors is needed. For example, the provision of an indoor open pack may influence the preference for pasture or an alternative outdoor area.

Despite numerous studies on the importance of pasture access for dairy cattle, little is known about what aspects of pasture are important to dairy cattle. For example, it is not known whether this preference for the outdoors is driven by a desire for more space, cooler air, softer surfaces, grass to graze, or some combination of these and other factors. When free-stall housed, mid-lactation dairy cows could choose between a large pasture or a smaller (i.e., 12 m^2^/cow) outdoor sand pack during the night in late summer, they spent around 90% of their time on pasture and only 1% on the sand pack (38). This preference could have been driven by the larger space that was available on the pasture compared to the sand pack, the ability to graze on pasture, or other factors. Similarly, Kismul et al. (39), showed that early-to-mid lactation cows with access to a small exercise pasture (0.2 ha grass-covered paddock with little herbage and provided *ad libitum* grass silage indoors) spent 44% of their time outside, compared to 81% for cows provided access to a production pasture (larger pasture with ample herbage and restricted access to grass silage indoors) (both groups were given 8.5 h/d outdoor access). Jørgensen et al. (40) showed that cows provided access to an exercise paddock (0.74 ha in size in a small forest) spent less time outdoors than did cows provided access to pasture (a total 2.8 ha in size that was used for strip grazing). These two latter studies were based on a single group of cows in each treatment and thus should be interpreted with caution given the lack of replication. When preference of free-stall housed cows for access to an outdoor pack was tested in summer and winter, cows spent 25% of the time outside in summer and only 2% in winter (41). Cows especially spent time outside during summer nights (50.0 ± 8.4%) rather than during summer days (3.3 ± 1.3 %) and generally avoided adverse weather (i.e., snow, strong wind, and/or low air temperatures) during the winter months. Haskell et al. (42) investigated the use of an outdoor concrete loafing area by free-stall housed cows when given the option during the day. These authors reported that the cows spent about 14% of their time outside on the concrete loafing area during the day in spring and summer, with the majority of this time being when the weather was sunny; cows rarely went outside in the rain. Except for cows given access to the production pasture in the study of Kismul et al. (39), feed was provided indoors in all studies such that cows could fulfill 100% of their nutritional needs without the need for grazing. Given that cow preference for pasture and alternative outdoor areas is affected by many factors, providing cows a choice to access the outdoors may be of particular importance. In addition, providing animals controllability over their environment likely enhances their welfare (43).

In the following section, we will outline how various dairy cattle behaviors (i.e., lying, standing, walking, feeding, social, and estrus behaviors) are influenced by different types of outdoor access, and how providing choice to access the outdoors can affect behavior.

## The Influence of Different Types of Outdoor Access on Spontaneous Dairy Cattle Behavior

### Lying, Standing, and Walking Behavior

Lying is a highly motivated behavior in dairy cows, with cows prioritizing lying over feeding after a period of deprivation of both behaviors (44). Heifers appear motivated to lie down for 12 to 13 h/d when housed in a tie stall (45); cows trained to push open a weighted gate to access an open deep-bedded lying area worked to maintain a lying time of 13 h/d (46).

Generally, cows housed on pasture have lower lying times compared to when housed indoors. For example, cows kept on pasture lay down for 10.9 vs. 12.3 h/d when housed in a free-stall barn (47). Other studies reported average daily lying times between 7.5 and 9.5 h/d for cows housed on pasture (48–50). Cows in free-stall barns typically lie down for 10–12 h/d (51–55), though large variation in average lying times exist between farms [between 9.5 and 12.9 h/d was reported by (52); between 8.7 and 13.2 h/d was reported by (55)]. Despite having lower lying times when housed on pasture, cows given the choice between pasture, and a free-stall barn generally chose to lie on pasture rather than indoors [e.g., (31, 56)], except during summer days when cows generally stay indoors [e.g., (31, 37)]. Cows are able to engage in a broader range of lying positions when housed on pasture, including lying flat on the side (57); the ability to adopt these positions may help explain cow preference for lying on pasture compared to the more restrictive lying environment of free-stalls. The surface type may also influence preference: when cows had a choice between an outdoor wood-chip area and pasture, they spent most of their lying time on the grass (58).

The lower daily lying times on pasture may be a consequence of time spent grazing, but to our knowledge no studies have attempted to disentangle whether indoor-housed cows provided access to pasture prefer to graze or to lie for long periods of time. Typical grazing times are difficult to estimate, given that these depend on several factors, including herbage height (59, 60), herbage allowance (61), and concentrate supplementation (62). Feral cattle spend from 6.8 to 13.0 h/d grazing [reviewed (63)], and Holstein–Friesian cows appear to spend about 9.2 h/d grazing (64, 65). Given these estimates, grazing time does not appear to be affected by lying time, but future research should investigate this. Higher lying times indoors may also be a consequence of boredom; an alternative explanation for the longer lying times reported in free-stalls compared to pasture is that cows are seeking refuge from the concrete standing surfaces elsewhere in the barn; soft, dry standing surfaces are rarely available indoors (66, 67). Lying stalls were designed to provide cows with a place for lying and not for standing. The ability of the cow to use the lying stall for standing is affected by the positioning of the neck-rail, with more aggressive positions (closer to the curb) increasing two foot standing (perching) in the stall (68). Hence, perching may be a result of cows looking for a soft place to stand, especially when the placement of the neck rail prevents cows from standing with all four feet in the stall (68). When housed in pens with rubber flooring in front of the feed bunk, cows spent less time perching, and standing fully in the free-stalls and less time lying down in the free-stalls, and more time standing idle at the feed bunk (66, 69). Boyle et al. (70), however, found no difference in lying time between cows housed in free-stall pens with concrete or rubber flooring, but found that cows housed on concrete stood more in the free-stalls compared to cows in pens with rubber flooring. In the latter case, cows stood more on the rubber flooring at the feed face, again suggesting that cows seek refuge from standing on hard surfaces. Taken together, these studies indicate that standing on a soft surface is important for dairy cattle.

These results may also help explain the partial preference of free-stall housed cows for outdoor bedded packs. Fregonesi et al. (71) showed that cows preferred to spend time both lying and standing fully in an indoor open bedded pack compared to free-stalls, potentially because of the less restrictive environment of the open pack. A study by Smid et al. (38) provided free-stall housed cows free access to an outdoor open sand pack or pasture during the night. Although the amount of time spent in each location differed, the proportion of time cows spent lying down outside was similar when given free access to a sand pack (55%) or pasture (52%), indicating that cows may find the outdoor pack comfortable for standing. Another study provided cows free access to an outdoor wood chip pack in summer and winter and found that in summer, 54% of the time that cows were outside was spent lying down. This again shows that cows preferred to stand on the outdoor pack for a significant amount of time. In winter cows spent little time outside and of the time spent outdoors only about 5% was spent lying down (41).

Cows generally walk more on pasture than in a free-stall barn [e.g., (72, 73)], likely because of the need to move while grazing. Exercise has been suggested to be positive for dairy cattle welfare (73), although the higher energy expenditure of cattle on pasture compared to zero-grazing systems may pose challenges (74). Pasture systems are often associated with lower body condition scores in dairy cattle [e.g., (75, 76)] emphasizing the need for good pasture management. The increased opportunities for exercise in outdoor areas compared to the generally more restrictive indoor housing environments may also provide benefits for animal welfare. This may be especially true in bedded packs as cows prefer to walk on softer materials such as rubber than on concrete flooring (67), potentially because they are more prone to falling and slipping on concrete (77).

### Feeding Behavior

Dairy cattle are able to utilize high roughage diets, but to maintain milk production and minimize body condition loss (75) many dairy cattle are fed more energy dense diets [often provided as a mixture of roughage and grain products, or as a total mixed ration [TMR; (78, 79)]. Ration formulation varies based on the nutritional demands of cows [see (80)]. On average, milk production increases when the diet is supplemented with grain (81, 82), and the perceived production benefits of feeding a mixed ration is one reason why cows are no longer kept on pasture (21).

It is important to distinguish between choice and forced outdoor systems. When cows were provided a choice between free-stall housing and pasture, they maintained much of their TMR intake, and increased their feeding rate as compared to when they were confined in the free-stall barn (31). A similar result was reported by Smid et al. (41) who provided cows access to an outdoor wood-chip pack and found that cows showed a small decline in their feeding time in summer, but no decrease in feeding time in winter, compared to when confined in the free-stall barn. When cows were provided access to pasture or an outdoor sand pack during the night, they had lower feeding times than when kept indoors day and night. However, regardless of the option to go outside during the night, cows maintained their feeding times indoors during the day (38). Overall, these studies indicate that, when provided a choice to access pasture or an alternative outdoor area, cows maintain much of their TMR intake. Cows can also maintain their intake (and milk production) when kept on pasture at night and indoors during the day, relative to cows kept permanently indoors (83).

An important difference between pasture and alternative outdoor areas is that only the former allows grazing. Cows given access to an alternative outdoor area are generally provided access to a TMR or partial mixed ration (PMR), feed sources that do not allow them to engage in typical “grazing” behavior. It is likely that cows are motivated to graze, but little work has addressed the importance of grazing for cow welfare. The inability to engage in natural feeding behaviors is associated with the development of stereotypic and other abnormal behaviors in many animal species [e.g., pigs: (84), giraffe: (85), chimpanzee: (86); horses: (87)]. Stereotypic behaviors often resemble the behavior that is thwarted (84). When grazing, cattle roll their tongue around the grass to ingest it; this behavior resembles tongue playing or tongue rolling [i.e., “twisting and twirling with the tongue, either inside or outside the open mouth,” (88)], one of the most common stereotypies in cattle. As described by Beauchemin (89), cows fed a TMR use their lips to ingest feed, as opposed to their tongue to ingest long-stemmed forage or when grazing grass (90). In experimental settings, oral stereotypies in cattle were never observed on pasture (90–92), but were present in loose housing (91). Thus, the method of feed ingestion may be as important for animals as the goal itself (i.e., ingesting feed). Interestingly, in mountain breeds such as Brown Swiss and Simmental, tongue rolling is more prevalent compared to other cow breeds (93). The reason for this is unknown. Jerseys also seem to be especially orally motivated, showing a higher frequency of cross-suckling than Danish Red or Holstein–Friesian calves (94). The lower prevalence of tongue rolling in other breeds does not necessarily indicate that they are less motivated to graze or to obtain roughage.

Prior experience may play an important role in determining the preference for pasture (11). Naïve heifers grouped with cows that had experience with grazing had a lower latency to graze compared to groups consisting of only naïve heifers. Differences in grazing behavior between the treatments were found only for the first hour after pasture introduction, however (95). Heifers that had experience with pasture spent less time grazing, but more time ruminating, compared to heifers with no experience with pasture. This potentially indicates more efficient grazing behavior of the former (96), grazing itself may not be the only factor influencing the preference for pasture access. As pasture provides cattle with roughage, grazing is confounded with roughage consumption. Research in this area is again limited, but the development and frequency of stereotypies has been linked with feeding low amounts of roughage (97). Calves appear to prefer long over chopped hay (98), and work on beef cows found that they were highly motivated to obtain roughage, especially when kept on a low-roughage diet (99). Collectively, these results indicate that access to roughage and the manipulation of feed are important to cattle, as also suggested in a review (100) on the importance of straw for dairy cattle.

The time of day that cows spend feeding indoors is mainly determined by the time of fresh feed delivery (101). On pasture, cows feed mainly during the day, with intense grazing bouts at dawn and dusk (102–104). Cows housed on pasture often show synchronized feeding (105) and lying behavior (73, 106), which is thought to be positive for their welfare, perhaps especially so for more subordinate cows (107).

Given that milk production per cow has more than doubled in the last 40 years (108), selection for milk yield may cause high producing dairy cows to be especially motivated to consume a high energy ration like that which is normally provided indoors. Given the selection for high milk production and the correspondingly high energy requirements, it has been questioned if certain dairy genotypes are suitable to be housed exclusively on pasture (109). We encourage research to disentangle the importance of grazing, roughage and energy provision for dairy cattle. Given that cows are unable to perform grazing behavior in alternative outdoor systems, understanding the importance of grazing for dairy cattle welfare will also provide insight into the acceptability of providing cows these alternatives. Studies have investigated the effect of outdoor access on feeding behavior, but these studies have not reported effects on drinking behavior. Given that the stocking rate for drinking places is typically higher than that for feeding places (110), and competition around the drinker may be expected, future work on drinking behavior is required.

### Social Behavior

Social behavior includes positive and negative (i.e., agonistic) interactions. Positive interactions in cows have not been studied extensively but there is some evidence that allogrooming (i.e., social licking) is important (111, 112). In contrast, agonistic interactions between cows have been well-studied and consist of multiple forms of aggressive behavior, such as displacements, pushes and head butts (88, 113, 114). Housing is thought to play an important role in the frequency and display of these interactions (115).

In free-stall housing, competition for resources such as feeding and lying areas may pose challenges. It is well-known that increased stocking density leads to increased competition for access to the feeding area in free-stall housed dairy cattle [e.g., (116, 117)]. When given a choice, cows prefer to have greater inter-cow distances than what is normally available in indoor systems (118, 119). Tresoldi et al. (115) investigated social behavior in dairy heifers housed in either a free-stall barn or kept on pasture. When housed in free-stalls, heifers exhibited a 4-fold increase in the number of social interactions (allogrooming as well as agonistic interactions) compared to when housed on pasture, but the ratio of positive to negative interactions was the same in the two environments. Less space was available indoors than on pasture, leading the authors to suggest that the higher number of social interactions observed indoors was a consequence of a higher stocking density.

Similar observations have been made for other types of outdoor areas. Heifers on an outdoor wood-chip pack, provided an individual space allowance of 8 m^2^ on the pack and 6 m^2^ on the concrete feeding area, showed increased frequency of play behavior, including social play, compared to heifers housed inside a free-stall pen that provided 5.3 m^2^/heifer (120). These heifers also had a higher frequency of allogrooming, but showed no difference in the frequency of agonistic interactions. Schütz et al. (121) reported that a minimum of 6 m^2^ of space allowance per cow was needed on an off-wintering rubber pad during an 18 h stand off period to maintain daily lying times similar to that observed when cows were housed on pasture. When cows were provided less space (3 or 4.5 m^2^/cow), the reduction in both lying time and lying bout duration and frequency was thought to be due to increased agonistic behavior. Free-stall housed cows given access to an outdoor open wood-chip pack spent more time outside during the night with increasing outdoor space allowance (range of space allowances tested: 4–16 m^2^) (122). Interestingly, outdoor space allowance did not influence the number of displacements from a lying position that cows were engaged in on the outdoor pack. However, as the authors argue, this latter finding may be a consequence of cows having the opportunity to avoid agonistic interactions by moving indoors, particularly when outdoor space per cow declines. Another study (42) reported that, compared to high-ranking cows, low-ranking, free-stall housed cows used an outdoor concrete loafing area more during the pre-feeding and feeding period, suggesting that the use of the outdoors may in part be affected by social rank. There is also some preliminary evidence suggesting that cows housed on an out-wintering woodchip pad showed a higher synchrony in lying and feeding behavior than free-stall housed cows (123). These authors argued that increased synchrony may be a positive indicator of welfare, but the work should be viewed with caution given that there were only two replications per treatment.

As an increase in space allowance generally results in reduced interactions between cows, it follows that providing cows with an additional outdoor space will result in a decline in social interactions. However, there has been little experimental work looking at how much space cows require on an alternative outdoor area. The Canadian Dairy Cattle Code of Practice (124) states that resting areas in bedded-pack pens must provide 11 m^2^ per mature cow, but no justification is provided for this number. New research is required to investigate the space requirements of individual cows when provided different forms of outdoor access. Studies should include social rank when investigating cow preference, as social rank may play an important role in the preference of dairy cows for certain environments. In addition, the effect of the choice to go outdoors on social interactions should be investigated, especially on outdoor areas other than pasture that typically provide less space per cow.

### Estrus Behavior

The estrus cycle in dairy cows is, on average, 21 days in length (125), with estrus behavior expressed between 2 and 24 h (126). Estrus behavior in dairy cows can be divided into primary (i.e., standing to be mounted) and secondary signs (i.e., anogenital sniffing, chin resting, successful, and unsuccessful mounts) (127).

Free-stall systems where cows are for the most part continuously housed on concrete flooring (representing the vast majority of US dairy operations; (21) pose a challenge for estrus expression. Cows housed in free-stall barns with concrete flooring have fewer standing estrus events (128) and a lower frequency of standing to be mounted compared to cows housed on pasture (129). Similar results were found comparing concrete with other types of flooring; for example, cows had a lower duration of estrus as well as a lower frequency of mounting and standing to be mounted when kept on concrete compared to dirt flooring (130). The effects of rubber flooring in loose housing systems are variable; cows housed on rubber mats showed a higher frequency of mounting than when housed on concrete (77), but no beneficial effects on estrus behavior of rubber over concrete flooring were found by Boyle et al. (70). Differences in rubber quality may explain this difference (131, 132).

Vailes and Britt (133) suggested that cows may feel unsure of their footing on concrete and are therefore less inclined to perform estrus behaviors on this flooring. Concrete flooring has been linked with more slipping during mounting compared to pasture (128) or rubber flooring (77). In the latter study, 19 out of 23 mounts on a concrete floor were accompanied with collapsing or slipping. Little information is available regarding the effect of alternative outdoor areas on estrus behavior. Cows housed in a covered straw yard had more successful mounting attempts compared to cows housed in a free-stall (134), possibly because the straw flooring provided them with better footing. Indeed, when given a choice between concrete and dirt, cows in estrus spent more time on dirt than on concrete and preferred to mount other cows that were in estrus on dirt rather than on concrete (133). However, the latter study was conducted with individual cows that were given 30 min to interact with two tied cows, one on concrete and one on dirt; to our knowledge no research has examined preferences for different types of flooring during estrus in dairy cows housed under commercial conditions.

Concrete flooring can also increase the risk of lameness in dairy cows [e.g., (47, 135, 136)]. Lame cows may be less inclined to engage in estrus behaviors, especially if the flooring contributes to their pain. Lame cows have lower behavioral estrus expression than non-lame cows (137). In addition, falling and slipping when mounting can increase the risk of trauma and lameness.

Based on these results, housing systems with softer, high traction flooring such as pasture, dirt, or deep bedded packs may facilitate the expression of estrus behavior in dairy cows. Access to an outdoor area with better footing than is provided by concrete may be especially beneficial to cows in estrus.

## Concluding Remarks

Pasture can provide cows with an open area and a soft surface that allows the expression of grazing and facilitates the expression of lying, standing, walking, and estrus behaviors. In addition, keeping cows on pasture decreases negative social interactions between cows, potentially because cows on pasture engage in fewer encounters compared to when housed indoors. Alternatives to pasture include outdoor loafing areas (often with concrete flooring) or outdoor open bedded packs. Given challenges with concrete or other hard flooring in terms of lying, standing, and walking behavior, bedded packs may be more suitable than concrete loafing areas. Access to an outdoor open bedded pack can facilitate lying, standing and walking behavior and may also have positive effects on social behavior. The benefits of an outdoor bedded pack on estrus behavior warrants more research, but the available evidence shows that outdoor bedded packs can provide better footing than is available indoors, minimizing slips which can be beneficial for estrus behavior. Alternative outdoor areas assessed to date appear to be less attractive than pasture, perhaps because these areas do not provide the opportunity to graze. We encourage future research to investigate the importance of grazing for dairy cattle welfare. The motivation of dairy cows to access alternative outdoor areas should also be investigated. Given that cow preference for the outdoors depends on many internal and external factors, providing cows a choice between well-managed indoor and outdoor areas may be of particular importance.

## Author Contributions

All authors contributed equally to conceptualization. A-MS performed the literature search and wrote the initial version of the review. DW and MK provided critical revisions.

## Conflict of Interest

The authors declare that the research was conducted in the absence of any commercial or financial relationships that could be construed as a potential conflict of interest.

## Footnotes

^1^Djurskyddslag SFS 2018:1192. Available online at: http://rkrattsbaser.gov.se/sfst?bet=2018:1192 (accessed May 1, 2019).
